# Influence of Fermi arc states and double Weyl node on tunneling in a Dirac semimetal

**DOI:** 10.1038/s41598-017-03991-4

**Published:** 2017-06-22

**Authors:** Zhuo Bin Siu, Can Yesilyurt, Mansoor B. A. Jalil, Seng Ghee Tan

**Affiliations:** 1 Computational Nanoelectronics and Nanodevices Laboratory, National University of Singapore, Singapore, Singapore; 2 Data Storage Institute, Agency for Science, Reseach and Technology, Singapore, Singapore

## Abstract

Most theoretical studies of tunneling in Dirac and the closely related Weyl semimetals have modeled these materials as single Weyl nodes described by the three-dimensional Dirac equation ${\boldsymbol{H}}{\boldsymbol{=}}{{\boldsymbol{v}}}_{{\boldsymbol{f}}}\overrightarrow{{\boldsymbol{p}}}\cdot \overrightarrow{{\boldsymbol{\sigma }}}$. The influence of scattering between the different valleys centered around different Weyl nodes, and the Fermi arc states which connect these nodes are hence not evident from these studies. In this work we study the tunneling in a thin film system of the Dirac semimetal Na_3_Bi consisting of a central segment with a gate potential, sandwiched between identical semi-infinite source and drain segments. The model Hamiltonian we use for Na_3_Bi gives, for each spin, two Weyl nodes separated in *k*-space symmetrically about *k*_*z*_ = 0. The presence of a top and bottom surface in the thin film geometry results in the appearance of Fermi arc states and energy subbands. We show that (for each spin) the presence of two Weyl nodes and the Fermi arc states results in enhanced transmission oscillations, and finite transmission even when the energy falls within the *bulk* band gap in the central segment respectively. These features are not captured in single Weyl node models.

## Introduction

The Dirac semimetal (DSM)^1–4^ is a topologically non-trivial state which has attracted much attention recently. Similar to the topologically protected surface states of the more well-established three-dimensional topological insulators (TIs)^5, 6^, the low energy dispersion relations of DSMs take the form of Dirac cones. Differing from the 3D TIs where there are odd numbers of Dirac cones within the Brillouin zone and the linear dispersion of the Dirac fermion Hamiltonian holds only in two dimensions for the surface states, the Weyl nodes in the *bulk* states of DSMs disperse linearly in all three spatial dimensions and appear in pairs. One Weyl node of each pair is a source of Berry curvature while the other node is a Berry curvature sink. Fermi arcs linking the two members of each pair emerge when a bulk DSM is truncated and a surface introduced perpendicular to the *k* space separation between the Weyl nodes. These Weyl nodes are topologically stable against perturbations which preserve the translational symmetry. To date, two materials, Cd_3_As_2_^7–10^ and Na_3_Bi^11–13^ have been experimentally confirmed to host the DSM state.

Most of the existing studies on tunneling in DSMs and the closely related Weyl semimetals WSMs have focused on infinitely sized bulk DSM/WSM slabs in the *k* space vicinity around a single Weyl node^14–16^. This neglects the effect of inter-valley tunneling between multiple Weyl nodes. The influence of Fermi arc states on the tunneling process is also not evident in these models as the explicit appearance of Fermi arcs requires pairs of Weyl nodes separated in *k*-space to be considered, as well as the presence of surfaces perpendicular to the *k* space separation between the arcs.

In order to elucidate the influence of multiple Weyl nodes and Fermi arc states on the transport compared with single Weyl node models of DSM/WSM tunneling, we study the tunneling in thin films of the DSM Na_3_Bi^17–20^ in an experimental setup similar to that in previous works, most of which have focused on Klein tunneling. We study the transmission from a semi-infinite source segment made of a DSM thin film to a semi-infinite drain segment of the same DSM thin film through a central segment which is subjected to a potential.

We mode the Na_3_Bi using the Hamiltonian of refs 1 and 2

1 $$H={\varepsilon }_{0}(\overrightarrow{k})+(\begin{array}{cccc}M(\overrightarrow{k}) & A{k}_{+} & 0 & 0\\ A{k}_{-} & -M(\overrightarrow{k}) & 0 & 0\\ 0 & 0 & M(\overrightarrow{k}) & -A{k}_{-}\\ 0 & 0 & -A{k}_{+} & -M(\overrightarrow{k})\end{array})$$

where ${\varepsilon }_{0}={C}_{0}+{C}_{1}{\pi }_{z}^{2}+{C}_{2}{\pi }^{2}$ and $M(\overrightarrow{\pi })={M}_{0}-{M}_{1}{\pi }_{z}^{2}-{M}_{2}{\pi }^{2}$,^1, 2^ ${\pi }^{2}={\pi }_{x}^{2}+{\pi }_{y}^{2}$ and *π*_±_ = *π*_*x*_ ± *iπ*_*y*_. The *π*_*i*_ = (*k*_*x*_ − *A*_*i*_)s are the minimal coupling momenta with *A*_*i*_ being the *i*th component of the electromagnetic vector potential to account for the magnetic fields which we will be introducing later. *C*_0_, *C*_1_, *C*_2_, *M*_0_, *M*_1_ and *M*_2_ are material parameters for which we assumed the same values as in ref. 1 The Hamiltonian consists of two uncoupled blocks representing the spin up and spin down states. Since the spin up and spin down states are uncoupled we can study each of these spin states separately. Here we focus on the spin up states. (The corresponding results for the spin down states can be obtained from those for the spin up states by replacing *k*_*y*_ →− *k*_*y*_).

This Hamiltonian yields, for an infinite-sized homogeneous Na_3_Bi crystal, two Weyl nodes per spin located at *k*_*x*_ = *k*_*y*_ = 0, *k*_*z*_ = ±Δ*k*_*z*_/2. In the infinite crystal the dispersion in the vicinity of each Weyl node is linear, reminiscent of a Dirac cone and can be identified with a ‘valley’.

**Figure 1:**
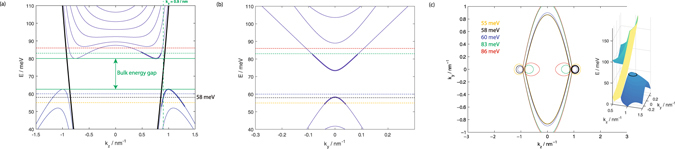
Panel (a) shows the dispersion relations at *k*_*y*_ = 0 for a 50 nm thick Na_3_Bi thin film. The bands in black correspond to the Fermi arc states. Each horizontal dotted line corresponds to the energy of the Equal Energy Contour (EECs) of the same colour in panel (c). The vertical dotted line denotes *k*_*z*_ = 0.9 nm^−1^, the value of *k*_*z*_ panel the *k*_*y*_ dispersion relations in panel (b) are plotted at. Panel (b) shows the dispersion relations at *k*_*z*_ = 0.9 nm^−1^. Panel (c) shows the EECs for the same thin film at various values of energy indicated in the figure legend. The inset shows the three-dimensional plot of the gapped Dirac with positive *k*_*z*_ with the black ring around the hole like states of the cone depicting the source states whose transmission we will study in this paper. The yellow sheet between the upper and lower cones corresponds to the Fermi arc states.

Panels (a) and (b) of Fig. 1 show that the introduction of the thin film geometry with infinite dimensions along the *y* and *z* directions and finite thickness *W* along the *x* direction lead to the formation of energy subbands due to the quantum confinement along the *x* direction. (The eigenspectrum was obtained by expanding the Schrödinger equation *H*|*ψ*〉 = |*ψ*〉*E* in the basis of the $\langle x|\psi \rangle =\sqrt{\frac{2}{W}}\,\cos (n\pi x/W)$ infinite potential well eigenstates and numerically diagonalizing the resulting matrix.)

The subbands, except for the specific bands indicated in black in panel (a) of the figure, are bulk subbands in the sense that there is significant particle density in the interior of the thin film away from the top and bottom surfaces. The cross sections of these bulk states on the *E* − *k*_*z*_ dispersion graphs of panel (a) correspond to the elliptical cross sections of the Dirac cones at small |*k*_*y*_| distributed symmetrically about *k*_*z*_ = 0 in panel (c). The subbands lead to the opening up of a bulk energy gap between the top of the hole bulk bands and the bottom of the particle bulk bands. In particular, sections of the lowest energy particle bulk band and the highest energy bulk band, highlighted by thicker lines in panels (a) and (b), correspond to the projections of the Weyl nodes in infinite-sized bulk Na_3_Bi. The presence of the top and bottom surfaces at *x* = ±*W*/2 results in the appearance of Fermi arc states^17, 18^, indicated in black in panel (a) of the figure, as well as in panel (c). These Fermi arc states differ from the bulk bands in that they are localized near the top or bottom surface of the thin film–for a given spin the arcs convex in the +*k*_*y*_ (−*k*_*y*_) direction are localized near the top (bottom) surface, and exist even inside the bulk energy gap^19^.

We note a few features in the EECs of Fig. 1 that will be needed later to explain the transmission profile. The figure corresponds to a Na_3_Bi thin film of thickness 50 nm. This will be the thickness of the thin films we study in the remainder of this paper. The finite thickness of the film leads to the opening of a bulk energy gap between 63 meV to 79 meV crossed only by the Fermi arc states. For the particular model Hamiltonian and parameter set of Na_3_Bi which we are employing, the elliptical cross sections of the Dirac cones at the two valleys shift inwards towards smaller values of |*k*_*z*_| as the energy increases. In order to connect with earlier single Weyl node studies of tunneling in WSM/DSM materials, we consider the energy and gate potential ranges when there is only to single propagating bulk band per valley and/or the Fermi arc state in the source and drain leads, and the central segment, and consider transmission from only the +ve *k*_*z*_ valley bulk states. (These states are highlighted in the black ring in the inset of panel (c) of Fig. 1b.) We set the lead energy at 58 meV as this gives a relatively wide range of gate potential in the central segment where only a single bulk band and/or the Fermi arc states are propagating. We consider cases where the interfaces between the central segment and the source/drain leads are parallel, as well as perpendicular to the *k* space separation between the Weyl nodes. We shall see that the presence of the two valleys as well as the Fermi arc states lead to the emergence of features not captured in earlier studies based on single Weyl nodes.

## Transmission

**Figure 2:**
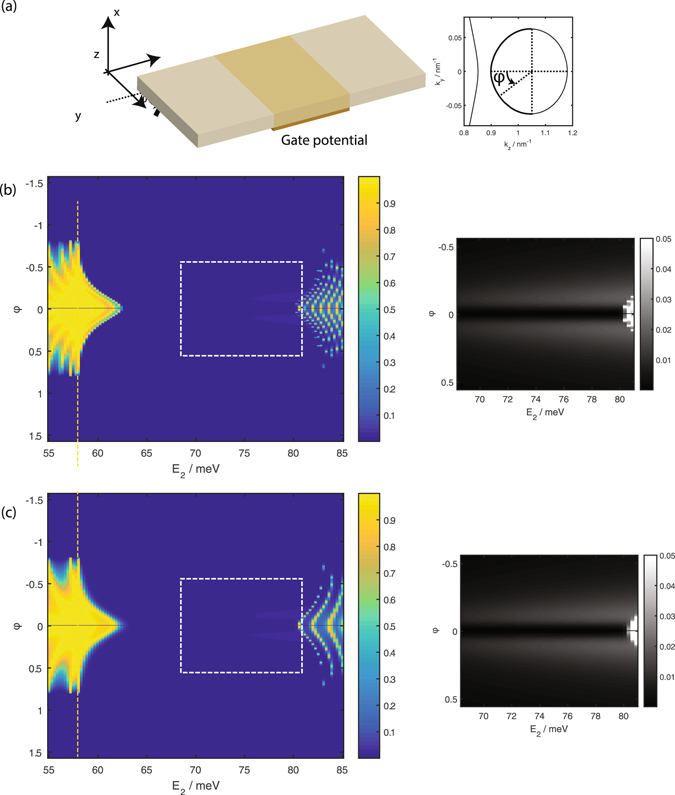
(**a**) A schematic of the tunneling setup consisting of a central segment subjected to a gate potential sandwiched between semi-infinite long source and drain leads. (**b**) and (**c**) show the transmission as a function of the angular coordinate *φ* along the positive *k*_*z*_ Dirac ‘cone’ (see inset of panel (a) for definition of *φ*), and *E*_2_ in the central segment for a central segment of length (**b**) 100 nm and (**c**) 50 nm. *φ* is roughly equal to the incident angle of the source current incident on the source-central segment interface as indicated in the main diagram of panel (a). The vertical yellow dotted line in panel (b) indicates the energy of the source and drain segments. The insets on the right of the main figures in panels (b) and (c) show the transmission within the *E*_2_ − *φ* range inside the dotted box of the main figures with the maximum transmission shown capped at 0.05 to emphasise the small but finite transmission due to tunneling to the Fermi arc states.

We first study the transmission from the positive *k*_*z*_ valley hole-like states at *E*_1_ = 58 meV source segment, through the central segment to the drain segment when the interfaces between the source and central segment, and between the central segment and the drain, are parallel to the *y* axis as shown in Fig. 2a. The system is hence translationally invariant along the *y* direction and *k*_*y*_ is conserved. The gate applies a potential which shifts the effective energy of the central segment *E*_2_ = *E*_1_ + *U* with respect to that of the source and drain leads. Panels (b) and (c) of Fig. 2 show the transmission as a function of *E*_2_ and the angular coordinate about the source Dirac cone *φ* for two different lengths of the central segment.

For *E*_2_ < *E* = 58 meV, the transmission is relatively high with a moderate amount of transmission oscillation for −*π*/2 < *φ* < *π*/2. As *E*_2_ increases between 58 meV to 63 meV the *φ* angular region where there is relatively high transmission tapers down to a point. This range of *E*_2_ corresponds to the transmission from the hole states in the source to the hole states in the central segment. The transmission is almost but not exactly 0 for values of *φ* for *E*_2_ ranging from 63 meV to 80 meV inside the bulk energy gap. As *E*_2_ increases from 58 meV there are two small angular ranges on either side of *φ* = 0 where there is a small but finite transmission which increases with *E*_2_. Finally as *E*_2_ increases above 80 meV the transmission becomes large again, with the *φ* angular range over which the transmission is large increasing with *E*_2_ until it spans the range from −*π*/2 < *φ* < *π*/2. The transmission for *E*_2_ > 80 meV corresponds to that from the hole states in the source to the particle states in the central segment. The transmission oscillations for *E*_2_ 80 meV are more prominent and occur with a shorter *E*_2_ periodicity compared to the fringes at *E*_2_ < 63 meV. Comparing now between panels (b) and (c) of the figure, we see that the qualitative features just noted are independent of the length of the central segment. The *E*_2_ periodicity of the transmission oscillations at *E*_2_ > 80 meV and *E*_2_ < 58 meV is shorter for the longer central segment (panel (b)) compared to the shorter one (panel (c)).

**Figure 3:**
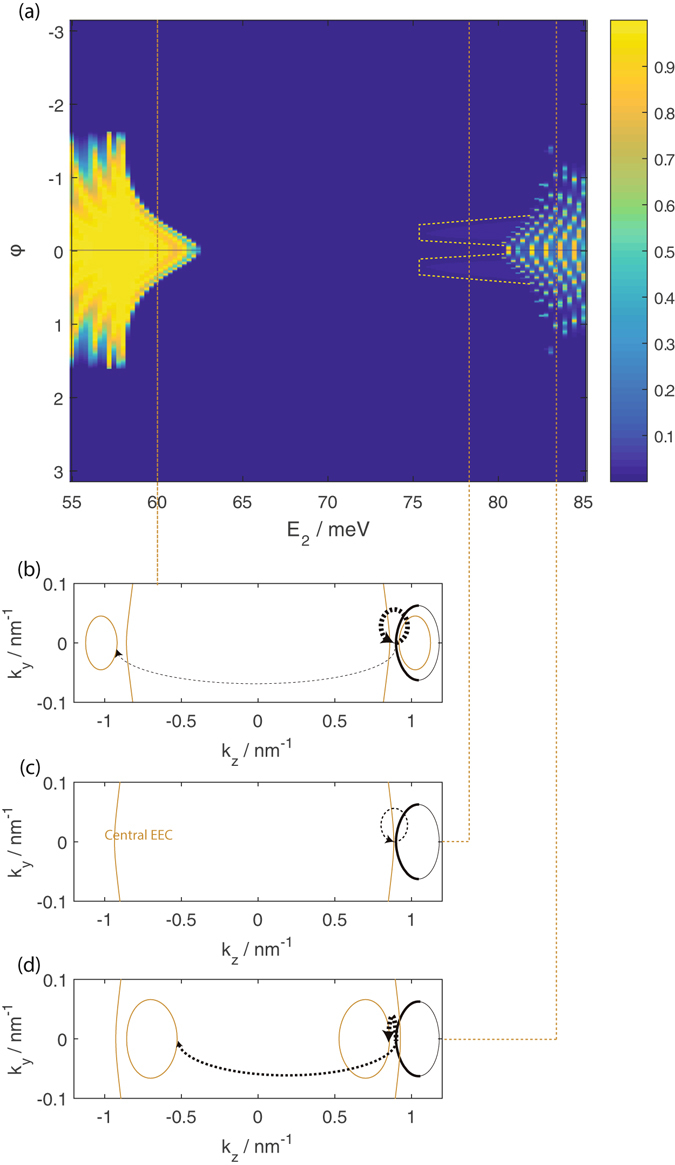
Panel (a) is the same plot of the transmission against angular coordinate as in Fig. 2(a). Panels (b) to (d) show the source (thick black lines) and central segment (thinner green lines) EECs at the indicated values of *E*_2_ in panel (a). The relative thickness of the dotted arrows pointing from points on the source EEC to the central segment EEC at *k*_*y*_ = 0 indicate the relative probabilities of the *k*_*y*_ = 0 central segment states propagating in the +*z* direction the source *k*_*y*_ = 0 state gets transmitted into. The thicker sections of the source EECs correspond to the source states which propagate in the +*z* direction.

These features may be explained by *k*_*y*_ conservation and the EEC profiles in the leads (i.e. the source and drain), and the central segments as shown in Fig. 3. The central segment wavefunction, ${{\rm{\Psi }}}^{(c)}(\overrightarrow{r})$ (the superscript (c) indicates that this is the central segment wavefunction) is, in general, a linear superposition of the various eigenstates with energy *E*_2_ and the given value of *k*_*y*_–

2 $${{\rm{\Psi }}}^{(c)}(x,y,z)=\sum _{j}{\psi }_{j}^{(c)}(x)\,\exp (i{k}_{y}y)\,\exp (i{k}_{z;j}z){c}_{j}\mathrm{.}$$

with weightings *c*_*j*_ for the *j*th central segment eigenstate. The relative tunnelling probabilities depicted in the figure indicate the relative magnitudes of the |*c*_*j*_|^2^ for the central segment *E*_2_ eigenstates for the central segment states which propagate in the +*z* direction. Thus, for example, the thinner arrows in panels (b) and (d) depict an inter-valley tunnelling process in which source states in the positive *k*_*z*_ valley tunnel into the negative *k*_*z*_ valley of the central segment.

The conservation of *k*_*y*_ restricts the angular range over which there is finite transmission to the range of *φ* in which there are propagating states for the values of source *k*_*y*_ which *φ* corresponds to. In general, the transmission probability from a bulk source state to a central segment Fermi arc state, while finite, is relatively low compared to the transmission probability to a central segment bulk state. This is largely due to the lack of spatial overlap between wavefunctions of the Fermi arc states, which are localized near either surface of the film, and the bulk states where significant probability density is found within the interior of the film. For *E*_2_ < 58 meV, the elliptical cross sections of the bulk ‘Dirac cones’ span a larger *k*_*y*_ range than the *k*_*y*_ range spanned by the bulk source EEC. The angular range of *φ* with high transmission is thus limited by the *k*_*y*_ range spanned by the source EEC and the transmission is relatively high over the entire range of −*π*/2 < *φ *< *π*/2 over which the source states propagate in the +*z* direction. Between 58 meV < *E*_2_ < 63 meV the *k*_*y*_ range spanned by the elliptical cross sections of the central segment Dirac hole cones shrink with increasing *E*_2_ to 0 at the bottom of the bulk energy gap. This leads to the narrowing of the range of *φ* with relatively high transmission to zero. We note from panel (b) that for *E*_2_ < 63 meV there is a small probability for a source state from the positive *k*_*z*_ valley to be transmitted to a central segment state at the negative *k*_*z*_ valley. (This probability is still significantly higher than that for transmission to a Fermi arc state.) This inter-valley transmission contributes to the oscillation of the transmission at fixed *φ* as the energy varies, due to the large *k*_*z*_ differences between the states involved.

The *E*_2_ range between 63 meV to 80 meV corresponds to the bulk gap in the central segment where the only propagating states are the Fermi arc states. The transmission of the bulk source states to the the central Fermi arc states gives a small but finite transmission over this *E*_2_ range.

As *E*_2_ increases beyond 80 meV the *φ* range with high transmission increases with energy as the *k*_*y*_ range spanned by the central segment EECs increases from zero to beyond the *k*_*y*_ range spanned by the source states. Compared to the *E*_2_ < 63 meV energy range, there is an increased probability of the positive *k*_*z*_ source states being transmitted to the other valley with negative *k*_*z*_. This may be due to the closer pseudospin alignment between the hole states in the positive *k*_*z*_ valley on the source side, and the particle states in the negative *k*_*z*_ valley on the central segment side, than the pseudospin alignment between the hole states in different valleys when *E*_2_ > 80 meV. This increased inter-valley transmission results in more prominent oscillations of the transmission at a fixed *φ* as the energy is varied beyond *E*_2_ > 80 meV compared to the oscillations at *E*_2_ < 63 meV.

(The reason why inter-valley transmission can result in more prominent transmission oscillations can be understood intuitively as follows. The transmission roughly corresponds to the carrier density at the drain side of the central-segment interface–if the transmission is zero then no carriers will be transmitted into the drain segment. The carrier density, by wavefunction continuity, is the same in both the drain as well as the central segment side of the interface. If we set the central-segment drain interface at *z* = *L* where *L* is the length of the central segment, then from Eq. 2 the carrier density at the interface is ${|{\psi }^{(c)}|}^{2}={\sum }_{a,b > a}2{\rm{Re}}(\exp (i({k}_{z;a}-{k}_{z;b})L){c}_{a}^{\ast }{c}_{b}\int {\rm{d}}x{\psi }_{a}^{\ast }(x){\psi }_{b}(x))$. A relatively large inter-valley transmission results in a significant contribution of terms where the *k*_*z*;*a*_ and *k*_*z*;*b*_ have opposite signs, so that the magnitude of the difference between the *z* wavevectors, |*k*_*z*;*a*_ − *k*_*z*;*b*_|, becomes large. The phase factor exp(*i*(*k*_*z*;*a*_ − *k*_*z*;*b*_)*L*) then varies rapidly with *E*_2_ as the *k*_*z*_s vary with energy, leading to the more prominent transmission oscillations. The dependence of the phase factor exp(*i*(*k*_*z*;*a*_ − *k*_*z*;*b*_)*L*) on *L* also explains why the transmission varies more rapidly for the longer segment in panel (b) of Fig. 2 than the shorter segment in panel (c) of the figure. In contrast if the largest wavevector difference magnitude |*k*_*z*;*a*_ − *k*_*z*;*b*_| between terms with significant |*c*_*a*_||*c*_*b*_| is smaller, the transmission varies less rapidly with *E*_2_, as we shall show when we discuss the transmission through a topologically trivial central segment shortly).

The inter-valley scattering and the Fermi arcs are absent in single Weyl node models. Hence, the enhanced transmission oscillations due to the inter-valley scattering, as well as the small angular ranges with small finite transmission occurring at energy ranges corresponding to the bulk energy gap of the central segment due to the Fermi arc states will not be captured in these models.

One may also ask the question of what happens when we replace the central segment with a topologically trivial material. One key distinction between a Dirac/Weyl semimetal and a topologically trivial normal material is that an infinite-sized bulk sample of the former has a gapless dispersion relation with the hole and particle bands touching at at least a single pair of Weyl nodes in three-dimensional *k*-space. For our Hamiltonian Eq. 1, the Weyl nodes occur at $\overrightarrow{k}=(\mathrm{0,0,}\pm \sqrt{\frac{{M}_{0}}{{M}_{1}}})$ in infinite-sized bulk Na_3_Bi. A topologically trivial material can therefore be obtained by replacing *M*_0_ with a number, ${M}_{0}^{^{\prime} }$ that has an opposite sign in order to eliminate the Weyl nodes and open up a bulk band gap. (We have checked that this does not result in a three-dimensional topological insulator).

**Figure 4:**
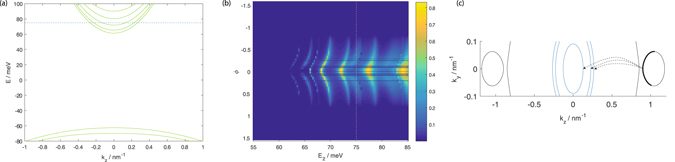
(**a**) shows dispersion relation of the model topologically trivial material obtained by setting ${M}_{0}^{^{\prime} }$ = 60 meVnm^2^ at *k*_*y*_ = 0. (**b**) shows transmission as a function of the angular coordinate *φ* along the positive *k*_*z*_ Dirac ‘cone’ and *E*_2_ in the central segment for a central segment of length 50 nm. (**c**) shows the central segment EEC at *E*_2_ = 75 meV, and the source EEC. The horizontal dotted line in panel (**a**) and vertical dotted line in panel (**b**) denote the *E*_2_ = 75 meV line.

Panel (a) of Fig. 4 shows the dispersion relation for a 50 nm thick film with ${M}_{0}^{^{\prime} }$ = 60 meVnm^2^ (*M*_0_ = −86.86 meVnm^2^ in Na_3_Bi). This value of ${M}_{0}^{^{\prime} }$ was chosen so that part of the bulk band gap lies within the *E*_2_ energy range in the transmission profiles considered here. Comparing panels (a) of Fig. 4 for the topologically trivial material with panel (a) of Fig. 1 for Na_3_Bi, one sees that the Fermi arc states spanning across the bulk energy gap in Na_3_Bi are now absent, so that there is now a bulk energy gap without any propagating states in the topologically trivial material. The projections of the Weyl nodes forming the two Dirac ‘cones’ in Na_3_Bi are absent as well, so that the bandstructure of the topologically trivial material now takes the form of roughly parabolic curves centered around $\overrightarrow{k}$ = 0 as can also be seen from the EEC curves in panel (c) of the figure. Panel (b) of the figure shows the transmission from a Na_3_Bi source to a Na_3_Bi drain through a 50 nm long, topologically trivial central segment. The transmission at *E*_2_ < 60 meV within the bulk band gap is almost but not exactly zero due to the contributions of evanescent states in the central segment. This transmission is, however, of a much smaller magnitude than the transmission through the Fermi arc states in the bulk band gap of Na_3_Bi and decays rapidly to zero away from the central segment particle band bottom at 60 meV. The transmission oscillates with *E*_2_ at *E*_2_ ranges above the particle band band bottom. The *E*_2_ periodicity of the transmission oscillations is larger than that in Fig. 1. In Na_3_Bi, intervalley tunnneling results in states propagating in the +*z* direction with both signs of *k*_*z*_ being present in the central segment so that the maximum magnitude of the *k*_*z*_ wavevector difference between the +*z* propagating states in Na_3_Bi is large. In contrast in the topologically trivial material there are neither Weyl nodes nor multiple valleys, so that the +*z* propagating states all have the same sign of *k*_*z*_ (see Panel (c)), and the maximum magnitude of the *k*_*z*_ wavevector difference is smaller.

Wheres Na_3_Bi is technically a Dirac semimetal because the spin up and spin down Weyl nodes are located at the same *k*-space points, the spin up and spin down states are uncoupled in our Hamiltonian Eq. 1 with the two partners of each pair of Weyl nodes located at different *k*-space points. The system considered here is therefore equivalent to two copies of a Weyl semimetal, one for each spin, where the Weyl nodes are seperated in *k*-space. Incidentally Fig. 4 also displays some of the qualitative trends in the transmission profile of a Dirac semimetal where both partners of a Weyl node pair are located at the same *k*-space point which may be obtained, for example, by setting *M*_0_ = 0. In such a DSM the hole and particle bands touch at a single *k*-space point so that whereas there is no bulk gap, there is neither inter-valley scattering nor Fermi arc states as well. The transmission profile would then look similar to that in Fig. 4 above the conductance band bottom at 60 meV with a transmission oscillating more slowly with *E*_2_ variation compared to a Weyl semimetal.

**Figure 5:**
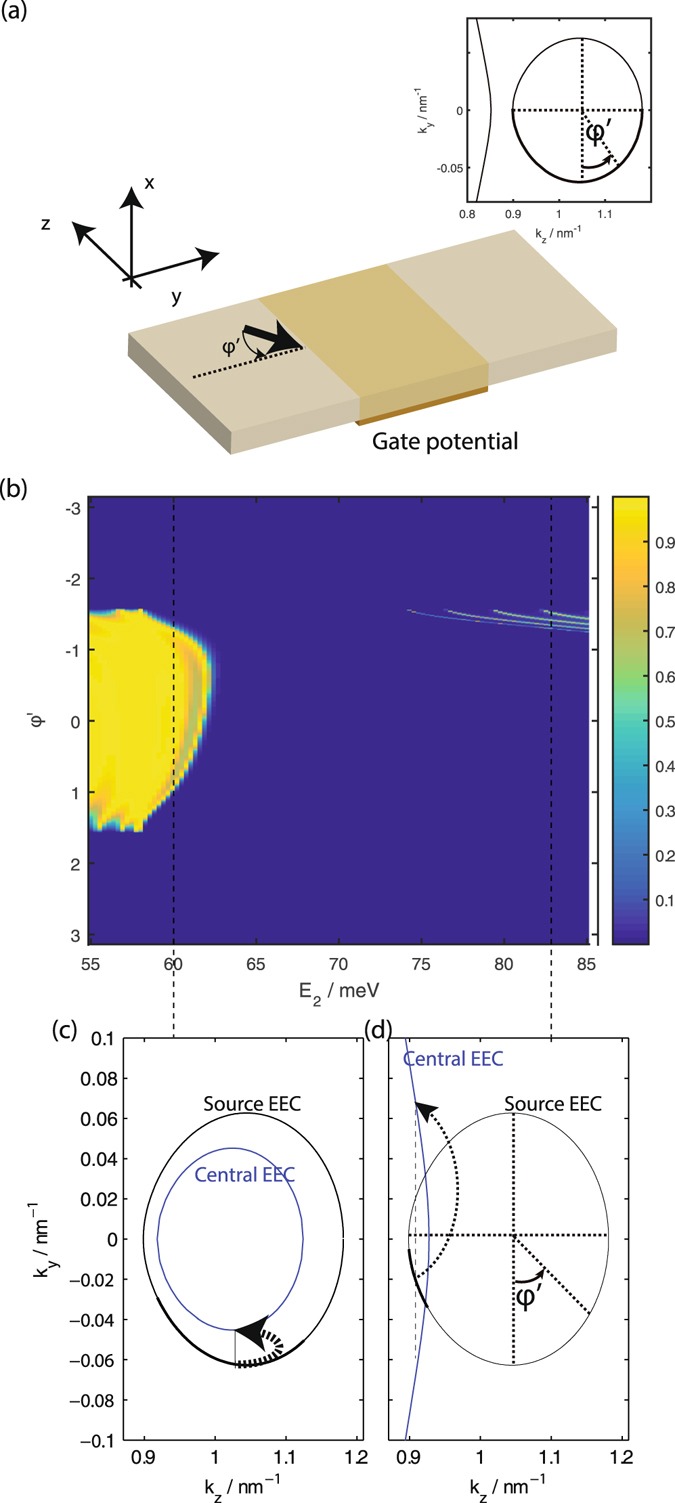
(**a**) The transmission as a function of the source angular coordinate *φ*′, and the central segment energy *E*_2_. *φ*′ is defined in the inset of the panel where the states propagating in the +*y* direction are indicated on the thicker line. (**b**) and (**c**) show the source (black) and central segment (blue) EECs at the *E*_2_ values indicated on the plot. The states on the source EEC propagating in the +*y* direction with ranges of *k*_*z*_ that overlap with the *k*_*z*_ range of states on the central segment propagating in the +*y* direction are indicated as thicker lines. The thickness of the arrows pointing from the source to the central segment EECs in (**b**) and (**c**) show the relative probability of transmission form the source states to the central segment states at the value of *k*_*z*_ indicated by the dotted lines.

We now turn our attention to the transmission from the source to the drain segment through a central segment subjected to a gate potential when the central segment-lead interfaces are along the *z* direction. In this case, *k*_*z*_ is conserved. The transmission profile is somewhat more difficult to compare directly against single Weyl-model models as the centers of the elliptical cross sections of the ‘Dirac cones’ here shift inwards towards smaller |*k*_*z*_| values with increasing energy (Fig. 1). This inwards shift leads to distinct features in the transmission profile, shown in Fig. 5. (Note that *φ*′ is defined differently from *φ* in the earlier figures in order for *φ*′ = 0 to correspond to normal incidence of the source wavefunctions on the source-central segment interfaces.)

Unlike the case where the lead-central segment interfaces are parallel to the *y* direction, the transmission for interfaces parallel to the *z* direction are not symmetrical about *φ*′ = 0. This asymmetry is due to the *k*_*z*_ shift of the center of the elliptical Dirac cone cross sections towards |*k*_*z*_| = 0 with increasing energy. This shift results in the central segment state with the same pseudospin direction as the source state at a given value of *k*_*z*_ having a slightly displaced value of *k*_*z*_. Another major difference is that there is zero transmission to the other valley here since the *k*_*z*_ ranges of the two valleys do not overlap. The lack of inter-valley transmission results in less prominent transmission oscillations with energy variation compared to Fig. 2

Similar to the interface along *y* case studied earlier, the transmission is relatively high for −*π*/2 < *φ*′ < *π*/2 for *E*_2_ < 58 meV. The angular range of *φ*′ with high transmission drops to zero as *E*_2_ is increased beyond 58 meV. This drop can be attributed to the combined effects of the shrinkage and displacement of the central EEC segment with increasing energy, both of which reduce the range of *k*_*z*_ overlap between the source and central segment EECs (panel (c) of Fig. 5). Between 64 meV < *E*_2_ < 75 meV the transmission is zero as there are no propagating central segment states within the *k*_*z*_ range spanned by the source EEC. As *E*_2_ increases beyond 75 meV the central segment Fermi arc starts to move into the *k*_*z*_ span of the source EEC, giving finite transmission again (panel (d)). The relatively large *k*_*y*_ difference between the central segment Fermi arc state and source bulk state at a given value of *k*_*z*_ results in more pronounced transmission oscillations with energy variation.

**Figure 6:**
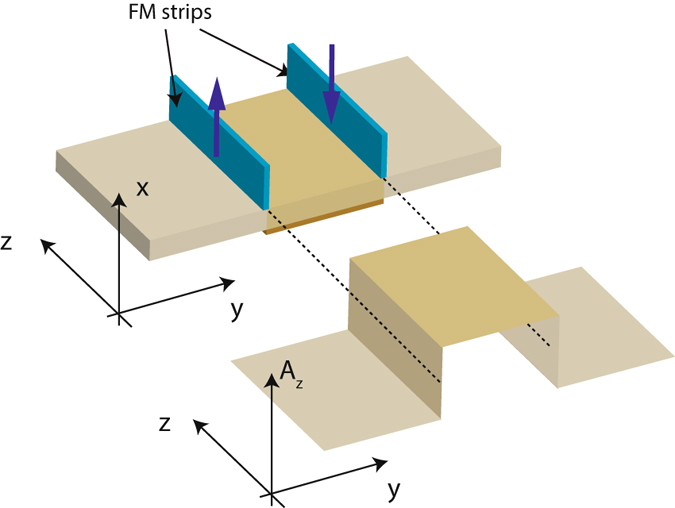
(**a**) A schematic of the tunneling setup with the inclusion of antisymmetrically magnetized FM strips, and (**b**) a plot of the corresponding *z* component of the EM vector potential which is proportional to the *k*_*z*_ shift.

The influence of the overlap between the momentum ranges perpendicular to the interface spanned by the lead and central segment EECs on the transmission profile suggests that the transmission profile can be manipulated by changing the overlap range. One way of achieving this is to introduce a shift of the EEC profile in momentum space by introducing antisymmetrically magnetized ferromagnetic (FM) strips to the interfaces between the leads and the central segment as shown in Fig. 6 ^21^. The FM strips produce localized magnetic fields which we model as Dirac delta functions. The vector potential in the central segment between the strips then takes the form of a step function. The effects of the magnetic field can be incorporated via the replacement of *k*_*i*_ → *π*_*i*_ ≡ (*k*_*i*_ − *A*_*i*_) = (*k*_*i*_ + *δk*_*i*_) where *A*_*i*_ is the electromagnetic vector potential. We choose the gauge such that the electromagnetic vector potential only has components along the *k* direction we want to displace. The FM strips hence lead to a translation of the EEC of the central segments in *k* space perpendicular to the direction of the FM magnetization.

**Figure 7:**
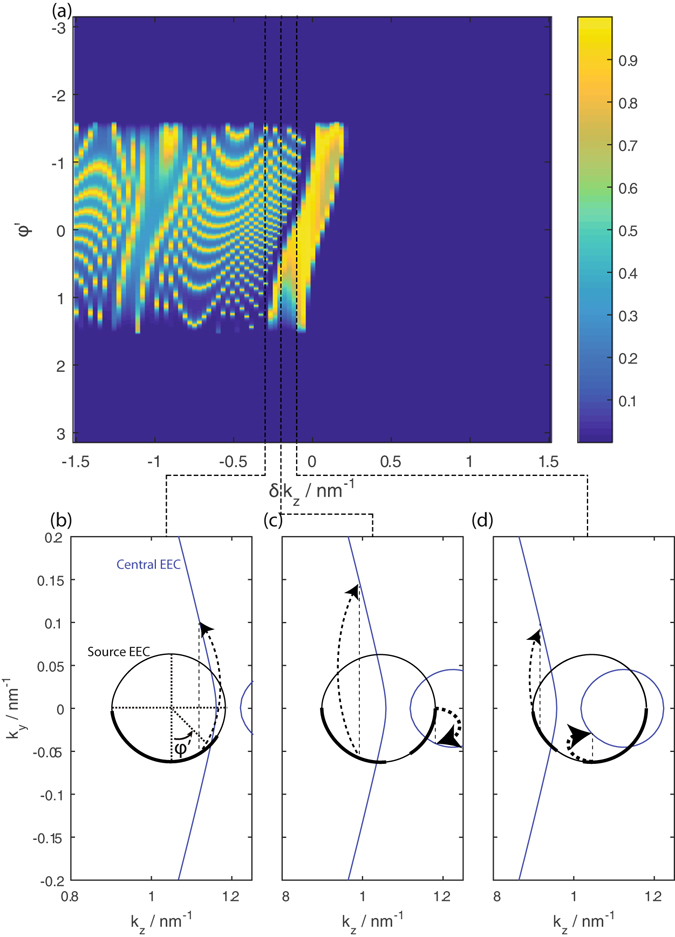
(**a**) The transmission as a function of the source angular coordinate *φ*′, and the central segment *k*_*z*_ shift *δk*_*z*_ at *E*_2_ = 60 meV. (**b**) to (**d**) show the source (black) and central segment (blue) EECs at the *δk*_*z*_ values indicated on the plot. The states on the source EEC propagating in the +*y* direction with ranges of *k*_*z*_ that overlap with the *k*_*z*_ range of states on the central segment propagating in the +*y* direction are indicated as thicker lines. Note the narrow *φ*′ ranges in (**c**) and (**d**) where part of the source EEC falls into the gap between the central segment bulk and Fermi arc states. The thickness of the arrows pointing from the source to the central segment EECs in (**b**) to (**d**) show the relative probability of transmission form the source states to the central segment states at the value of *k*_*z*_ indicated by the dotted lines.

Figure 7 shows the transmission from such a setup with fixed *E*_2_ = 60 meV and length 100 nm as a function of the FM strip induced *k*_*y*_ shift *δk*_*y*_ and angular coordinate *φ*′ around the source EEC.

The transmission is zero for large positive values of *δk*_*z*_ when the largest value of *k*_*z*_ for the drain propagating states at *k*_*y*_ = 0 on the bulk Fermi arcs are pushed beyond the smallest value of *k*_*z*_ on the source EEC, resulting in 0 overlap between the source and drain EECs. The transmission profile for smaller, and negative values of *δk*_*z*_ in the figure can be divided into three regime–a narrow band of *δk*_*z*_ range where the transmission is near unity and oscillates weakly, an even narrower band of 0 transmission as *k*_*z*_ decreases further, and a broad band of *k*_*z*_ range where the transmission shows significant oscillation with *δk*_*z*_.

The narrow band of *δk*_*z*_ with near unity transmission corresponds to the *k*_*z*_ range where *k*_*z*_ values of the source and drain bulk EECs overlap. (They overlap partially in panels (c) and (d) of Fig. 7.) The transmission is then contributed largely by the transmission from source to central segment bulk states, which occurs at a higher probability than the transmission from source bulk states to central segment Fermi arc states. Within this energy band, the transmission peaks for a given *φ* occur when *δk*_*z*_ compensates exactly for the *E*_2_ shift in the central segment Dirac cone cross sectional ellipse so that the pseudospin orientation between the source and central segment EECs at a given value of *k*_*z*_ are aligned.

As *δk*_*z*_ decreases we encounter the situation (panel (c) and (d)) where a narrow *φ* range on the source EEC falls into the *k*_*z*_ range lying between the largest value of *k*_*z*_ spanned by source bulk states and the smallest value of *k*_*z*_ spanned by the Fermi arc states. There is 0 transmission in this *φ* range since there are no propagating central segment states at the corresponding values of *k*_*z*_.

A further decrease in *δk*_*z*_ leads to more, and eventually, all of the *k*_*z*_ range spanned by the source EEC overlapping with that spanned by the central segment Fermi arc EECs. The relatively large *k*_*y*_ vector differences between the source and central segment states propagating in the +*z* direction lead to the more pronounced transmission oscillations.

## Conclusion

In this work, we studied the transmission of the hole states of the positive *k*_*z*_ valley in Na_3_Bi thin film from a source lead through a central segment with a gate potential to an identical drain lead. The finite thickness of the thin film results in the appearance of Fermi arc states as well as energy subbands in the dispersion relation. We considered the two cases where the interfaces between the leads and the central segment are (i) perpendicular and (ii) parallel to the *k* space separation in the *k*_*z*_ direction between the Weyl nodes. We saw that in the first case, inter-valley scattering gives rise to pronounced transmission oscillations with the variation of the central segment length and gate potential while the Fermi arc results in finite transmission even when the energy falls within the central segment bulk energy gap. Both of these features are not captured by earlier studies based on models which consider only a single Weyl node. We also saw that for interfaces parallel to the *z* direction, the Fermi arc states result in a finite transmission even when there is zero transverse momentum overlap between the source and central segment *bulk* states. This transmission can be modulated by the introduction of thin ferromagnetic strips at the central segment-lead interfaces which induce an electromagnetic vector potential that translate the central segment equal energy contours in *k* space along the direction of the vector potential.

We note that the Hamiltonian Eq. 1 consists of two uncoupled blocks for spin up and spin down respectively, each of which gives the same transmission profile. The key qualitative trends observed in this work–that the Fermi arc states give a small but finite transmission for central segment energies falling inside the bulk energy gap, and that inter-valley tunnelling results in pronounced transmission oscillations–are independent of the spin orientations of the eigenstates. These trends will still be observed in a *non* spin-degenerate Weyl semimetal thin film. (The exact numerical values of the energies and incidence angles where transmission peaks occur will of course depend on the detailed spin/pseudospin orientations of the eigenstates.) In particular, if the coupling between the two spin channels is weak or non-existent each spin channel can be treated separately so that the rough outlines of the transmission profile may be obtained by first translating the profiles here in energy to account for the energy difference of the Weyl nodes for each channel and then summing up the contributions of the two spin channels.
